# Supplementary material for: Altered regulation of mesenchymal cell senescence in adipose tissue promotes pathological changes associated with diabetic wound healing
Source: Commun Biol. 2022 Apr 5;5:310. doi: 10.1038/s42003-022-03266-3 (PMC8983691; doi:10.1038/s42003-022-03266-3)
Supplement: Supplementary file 2 — Supplementary Information [file 42003_2022_3266_MOESM2_ESM.pdf]

Supplementary File for

Diabetic wound impaired injury-induced transient senescence in adipose tissue that promotes the normal wound healing process

Authors: Arisa Kita MD[1], Yuki Saito PhD[2]*, Norihiro Miura[3], Maki Miyajima PhD[3], Sena Yamamoto[3], Tsukasa Sato[3], Takatoshi Yotsuyanagi MD,PhD[1], Mineko Fujimiya MD, PhD[2], and Takako S. Chikenji PhD [2,3]*

Affiliations:
1. Department of Plastic and Reconstructive Surgery, Sapporo Medical University, Sapporo, Japan
2. Department of Anatomy, Sapporo Medical University School of Medicine, Sapporo , Japan
3. Graduate School of Health Sciences, Hokkaido University, Sapporo, Japan.

***Corresponding authors:**
Takako S. Chikenji
Address: North12 West5, Kitaku, Sapporo, 060-0812, Japan
Telephone: 011-706-3382 FAX: 011-706-3382
Email: chikenji@pop.med.hokudai.ac.jp

Yuki Saito
Address: South1 West17, Chuoku, Sapporo, 060-8556, Japan
Telephone: 011-611-2111 FAX: 011-618-4288
Email: yuki.saito@sapmed..ac.jp

Supplementary Figures

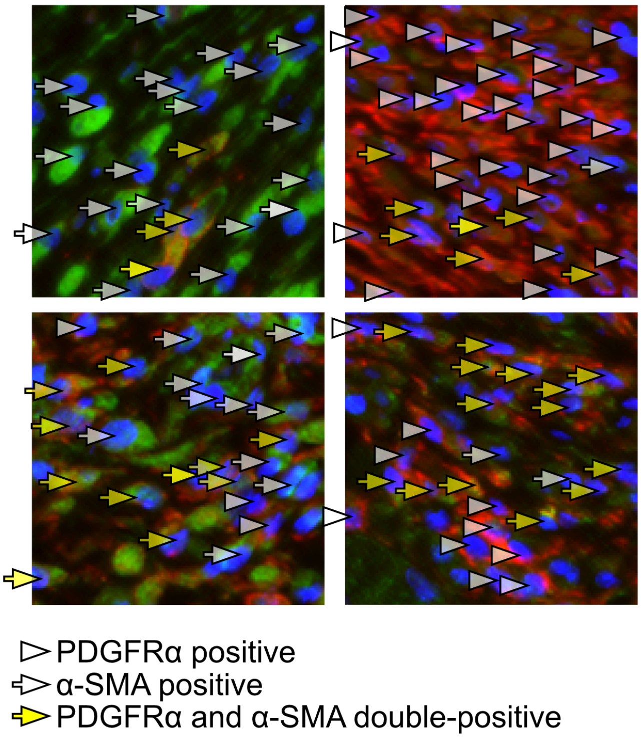

Supplementary Figure 1. Representative images of the definition of PDGFRα and α-SMA positive cells

White arrowheads indicate PDGFRα-positive cells, white arrows indicate α-SMA–positive cells, and yellow arrows indicate cells positive for both PDGFRα and α-SMA cells.

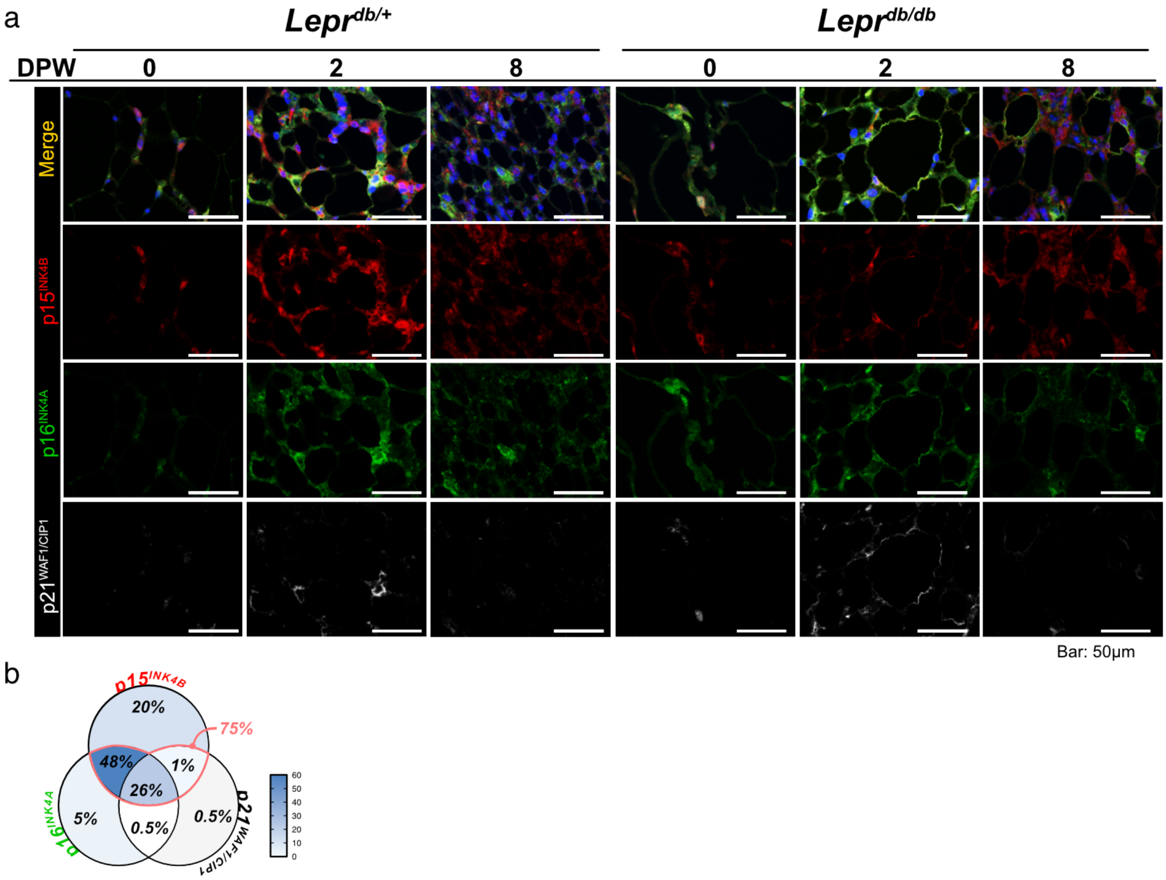

Supplementary Figure 2. p15^{INK4B}, p16^{INK4A}, and p21$^{WAF1/CIP1}$ expression in subcutaneous adipose tissue during wound healing in diabetic mice

(a) Representative images of p15INK4B, p16INK4A, and p21WAF1/CIP1 immunostaining of adipose tissue at pre-wounding, 2 DPW, and 8 DPW in Leprdb/+ and Leprdb/db mice and (b) Venn diagram showing proportions of cells positive for any or all of p15^{INK4B}, p16^{INK4A}, and p21$^{WAF1/CIP1}$.

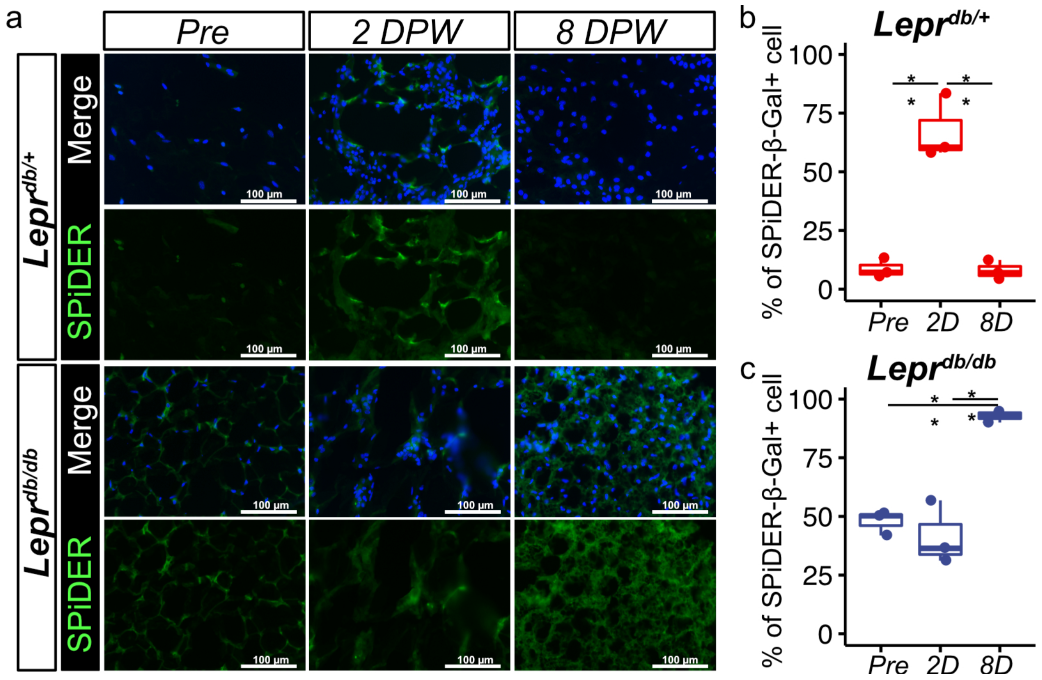

Supplementary Figure 3. SPiDER-β-Gal staining in subcutaneous adipose tissue during wound healing in Lepr$^{db/+}$ and Lepr$^{db/db}$ mice

(a) Representative images of SPiDER-β-Gal staining of adipose tissue, and **(b–c)** quantitative data for the percentage of SPiDER-β-Gal–positive cells (n = 3 for each group) at pre-wounding, 2 DPW, and 8 DPW. Quantitative data are presented as box-and-whisker plots with IQRs and 1.5 times the IQR. p-values were determined using the Tukey method for one-way ANOVA (*$p < 0.05$ and **$p < 0.001$).

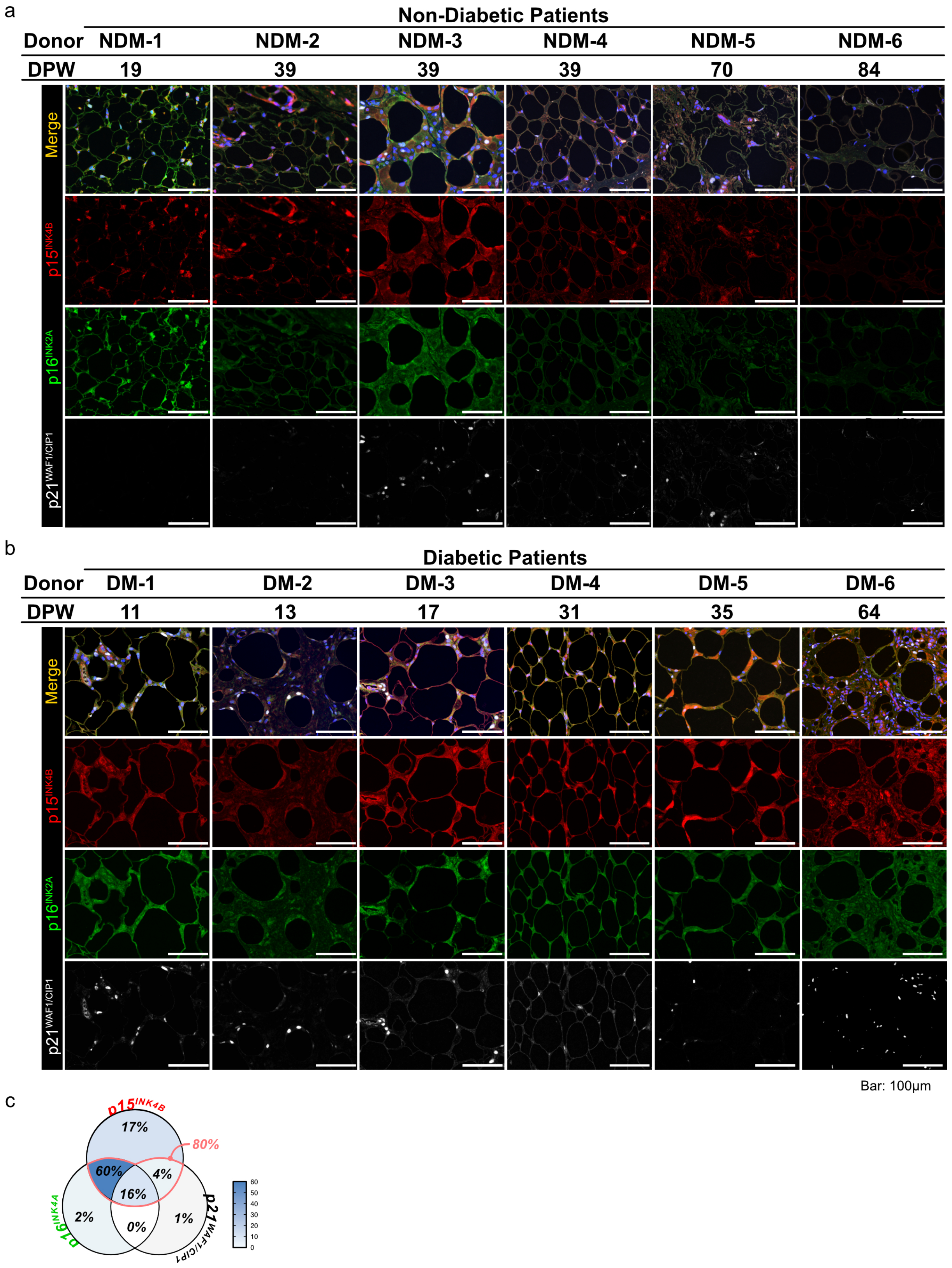

Supplementary Figure 4. p15^{INK4B}, p16^{INK4A}, and p21$^{WAF1/CIP1}$ expression in subcutaneous adipose tissue during wound healing in diabetic patients

(a–b) Representative images showing adipose tissue following immunostaining for p15^{INK4B}, p16^{INK4A}, and p21$^{WAF1/CIP1}$. Tissue samples were collected tissue during wound healing in

diabetic or non-diabetic patients, and (c) Venn diagram plot showing proportions of cells positive for any or all of $p15^{INK4B}$, $p16^{INK4A}$, and $p21^{WAF1/CIP1}$.

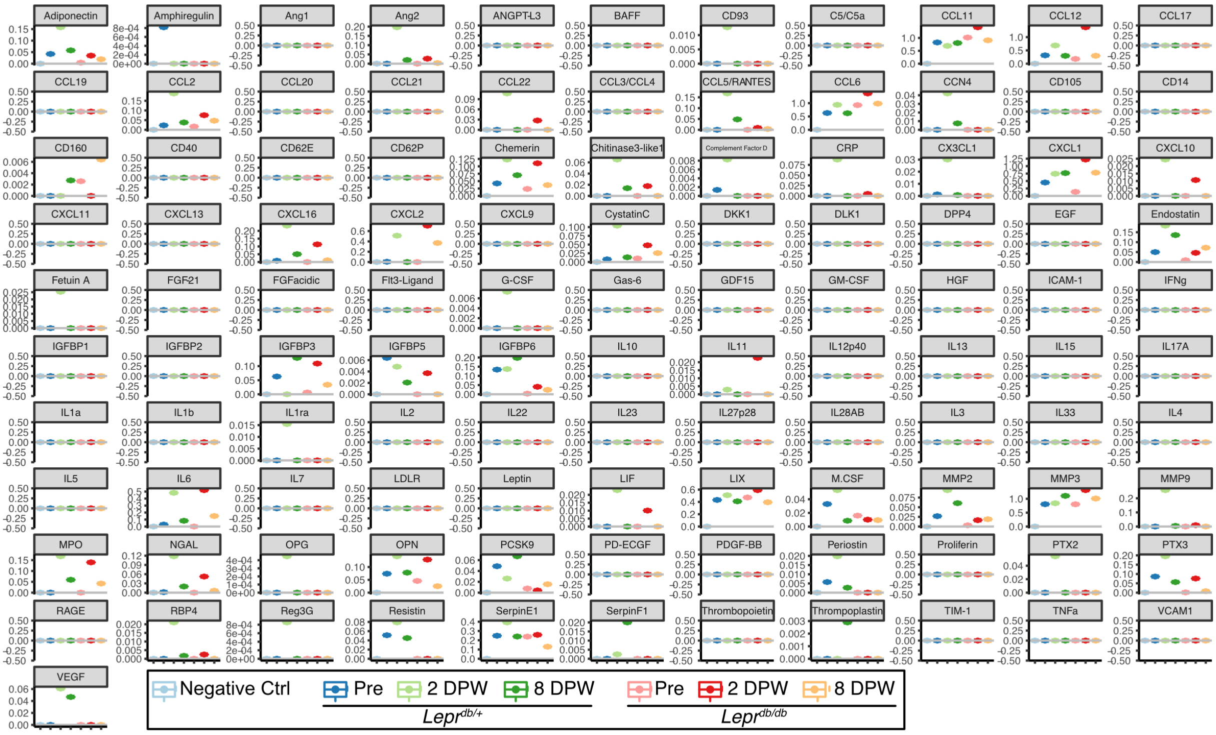

Supplementary Figure 5. Quantitative data from Proteome profiler antibody array

Quantitative data of Proteome profiler antibody array of SASP-containing culture media collected from organ culture of adipose tissue at pre-wounding, 2 DPW, and 8 DPW.

Supplementary Table.1 Specific primer sequence used for real-time PCR

Gene	Forward	Reverse	Size	Ascension Number
Cdkn2b	5' AATAACTTCCTACGCATTTTCTGC 3'	5' CCCTTGGCTTCAAGGTGAG 3'	64	NM_007670.4
Cdkn1a	5' GTACTTCCTCTGCCCTGCTG 3'	5' AGAAGACCAATCTGCGCTTG 3'	183	NM_007669.5
Trp53	5' AGCATCTTATCCGGGTGGAAG 3'	5' CCCATGCAGGAGCTATTACACA 3'	157	NM_011640.3
Serpine1	5' CCTCTTCCACAAGTCTGATGGC 3'	5' GCAGTTCCACAACGTCATACTCG 3'	119	NM_008871.2
Serpine2	5' ACTGTCTGCCATCATCCCTCAC 3'	5' GTAATGCCAAGGGCTTTCAGTGG 3'	150	NM_009255.4
IL6	5' TACCACTTCACAAGTCGGAGGC 3'	5' CTGCAAGTGCATCATCGTTGTTC 3'	116	NM_031168.2
Tgfb1	5' GCCTGAGTGGCTGTCTTTTGA 3'	5' CACAAGAGCAGTGAGCGCTGAA 3'	101	NM_011577.2
Pdgfra	5' GCAGTTGCCTTACGACTCCAGA 3'	5' GGTTTGAGCATCTTCACAGCCAC 3'	159	NM_001083316.2
Acta2	5' ACGCTGAAGTATCCGA 3'	5' CATTTTCTCCCGGTTGG 3'	162	NM_007392.3
Gapdh	5' AGGTCGGTGTGAACGGATTTG 3'	5' TGTAGACCATGTAGTTGAGGTCA 3'	123	NM_001289726.1
Atcb	5' CATTGCTGACAGGATGCAGAAGG 3'	5' TGCTGGAAGGTGGACAGTGAGG 3'	138	NM_007393.5
