# Supplementary material for: Altered regulation of mesenchymal cell senescence in adipose tissue promotes pathological changes associated with diabetic wound healing
Source: Commun Biol. 2022 Apr 5;5:310. doi: 10.1038/s42003-022-03266-3 (PMC8983691; doi:10.1038/s42003-022-03266-3)
Supplement: Supplementary file 3 — Description of Additional Supplementary Files [file 42003_2022_3266_MOESM3_ESM.pdf]

Description of Additional Supplementary Files

File name: Supplementary Data 1
Description: The source data behind the graphs.
