# Supplementary material for: Altered regulation of mesenchymal cell senescence in adipose tissue promotes pathological changes associated with diabetic wound healing
Source: Commun Biol. 2022 Apr 5;5:310. doi: 10.1038/s42003-022-03266-3 (PMC8983691; doi:10.1038/s42003-022-03266-3)
Supplement: Supplementary file 5 — Reporting Summary [file 42003_2022_3266_MOESM5_ESM.pdf]

nature research

Corresponding author(s): Takako S. Chikenji
Yuki Saito

Last updated by author(s): Feb 21, 2022

Reporting Summary

Nature Research wishes to improve the reproducibility of the work that we publish. This form provides structure for consistency and transparency in reporting. For further information on Nature Research policies, see our Editorial Policies and the Editorial Policy Checklist.

Statistics

For all statistical analyses, confirm that the following items are present in the figure legend, table legend, main text, or Methods section.

n/a	Confirmed	
☐	☒	The exact sample size (*n*) for each experimental group/condition, given as a discrete number and unit of measurement
☐	☒	A statement on whether measurements were taken from distinct samples or whether the same sample was measured repeatedly
☐	☒	The statistical test(s) used AND whether they are one- or two-sided<br>*Only common tests should be described solely by name; describe more complex techniques in the Methods section.*
☒	☐	A description of all covariates tested
☐	☒	A description of any assumptions or corrections, such as tests of normality and adjustment for multiple comparisons
☐	☒	A full description of the statistical parameters including central tendency (e.g. means) or other basic estimates (e.g. regression coefficient) AND variation (e.g. standard deviation) or associated estimates of uncertainty (e.g. confidence intervals)
☐	☒	For null hypothesis testing, the test statistic (e.g. *F*, *t*, *r*) with confidence intervals, effect sizes, degrees of freedom and *P* value noted<br>*Give P values as exact values whenever suitable.*
☒	☐	For Bayesian analysis, information on the choice of priors and Markov chain Monte Carlo settings
☐	☒	For hierarchical and complex designs, identification of the appropriate level for tests and full reporting of outcomes
☐	☒	Estimates of effect sizes (e.g. Cohen's *d*, Pearson's *r*), indicating how they were calculated

Our web collection on statistics for biologists contains articles on many of the points above.

Software and code

Policy information about availability of computer code

Data collection	Histological and immunohistochemical data collection were conducted fluorescence microscope BZ-X700 (Keyence Corp., Osaka, Japan).<br>Immunoblotting data collection were conducted ChemiDOC MP (BioRad) and Image Lab Software (BioRad).<br>Image J software, version 1.5 (National Institutes of Health, Bethesda, MD, USA) was used for data analysis.
Data analysis	Statistical analyses were performed using R (The R Foundation for Statistical Computing, Vienna, Austria). Statistical significance between two groups was determined using an unpaired t-test. A one-way or two-way analysis of variance (ANOVA) was conducted to assess differences among three or more groups. Pairwise comparisons were made only when the ANOVA test identified a statistical significance. p-values for multiple comparisons were adjusted using the Tukey method. Statistical analyses were performed using EZR, which is a graphical user interface for R.<br>Quantitative data are presented as either the mean±standard error of the mean (SEM) or median with interquartile range (IQR) and 1.5 × IQR. Box-and-whisker plots and bar plots were generated using ggplot2, a plotting system for R based on The Grammar of Graphics (The R Foundation for Statistical Computing, Vienna, Austria). The R packages FactoMineR and factoextra were used to generate heat maps, Ward's hierarchical agglomerative clustering, and principal component analyses.

For manuscripts utilizing custom algorithms or software that are central to the research but not yet described in published literature, software must be made available to editors and reviewers. We strongly encourage code deposition in a community repository (e.g. GitHub). See the Nature Research guidelines for submitting code & software for further information.

Data

Policy information about availability of data

All manuscripts must include a data availability statement. This statement should provide the following information, where applicable:

- Accession codes, unique identifiers, or web links for publicly available datasets
- A list of figures that have associated raw data
- A description of any restrictions on data availability

All data generated or analysed during this study are included in this published article and its supplementary information files (supplementary data 1).

Field-specific reporting

Please select the one below that is the best fit for your research. If you are not sure, read the appropriate sections before making your selection.

☒ Life sciences ☐ Behavioural & social sciences ☐ Ecological, evolutionary & environmental sciences

For a reference copy of the document with all sections, see nature.com/documents/nr-reporting-summary-flat.pdf

Life sciences study design

All studies must disclose on these points even when the disclosure is negative.

Sample size	The established scientific standard of n≥3 was applied throughout this study.
Data exclusions	Mice were only excluded from the study if they had visible wounds from fighting.
Replication	All experiments were independently repeated using at least three mice per experimental group.
Randomization	We did not use any specific method of randomization to determine how animals were allocated to experimental groups.
Blinding	The investigators were not blinded to allocations during experiments or outcome assessments, although they were blinded during the histological assessment.

Reporting for specific materials, systems and methods

We require information from authors about some types of materials, experimental systems and methods used in many studies. Here, indicate whether each material, system or method listed is relevant to your study. If you are not sure if a list item applies to your research, read the appropriate section before selecting a response.

Materials & experimental systems

n/a	Involved in the study	
☐	☒	Antibodies
☒	☐	Eukaryotic cell lines
☒	☐	Palaeontology and archaeology
☐	☒	Animals and other organisms
☐	☒	Human research participants
☒	☐	Clinical data
☒	☐	Dual use research of concern

Methods

n/a	Involved in the study	
☒	☐	ChIP-seq
☒	☐	Flow cytometry
☒	☐	MRI-based neuroimaging

Antibodies

Antibodies used

Antibody/Clone/Dulution/Source/Cat. No.
Rabbit anti-p15/polyclonal/ 1:500/ abcam/ ab53034
Rabbit anti–PDGFR-a /D1E1E/1:500-1000/Cell Signaling Technology/ 3274S
Rabbit anti–a-SAM /D4K9N/1:500/Cell Signaling Technology/ 19245S
Rabbit anti–phosphpa-histone H2A.X(Ser139)/20E3/1:480/Cell Signaling Technology/ 9718S
Rabbit anti–p16INK4A/5F22/1:200/ZooMAb/Sigma-Aldrich/ZRB1437
Rabbit anti–p21/5G7/1:1000/ZooMAb/Sigma-Aldrich/ZRB1141

Validation

Antibody/Clone/Dulution/Source/Cat. No.
Rabbit anti-p15: https://www.abcam.co.jp/p15-ink4b-antibody-ab53034.html
Rabbit anti–PDGFR-a : https://en.cellsignal.jp/products/primary-antibodies/pdgf-receptor-a-d1e1e-xp-rabbit-mab/3174
Rabbit anti–a-SAM : https://en.cellsignal.jp/products/primary-antibodies/a-smooth-muscle-actin-d4k9n-xp-rabbit-mab/19245
Rabbit anti–phosphpa-histone H2A.X(Ser139): https://en.cellsignal.jp/products/primary-antibodies/phospho-histone-h2a-x-

ser139-20e3-rabbit-mab/9718
Rabbit anti-p16INK4A: https://www.sigmaaldrich.com/JP/ja/product/sigma/zrb1437
Rabbit anti-p21: https://www.sigmaaldrich.com/JP/ja/product/sigma/zrb1141

Animals and other organisms

Policy information about studies involving animals; ARRIVE guidelines recommended for reporting animal research

Laboratory animals	Male C57BLKS/JIar-+Leprdb/+Leprdb (Leprdb/db), male C57BLKS/JIar-m+/+Leprdb (Leprdb/+), and male C57BL/6 mice (age>11 weeks; Sankyo Lab Service, Tokyo, Japan)
Wild animals	This study did not use wild animals.
Field-collected samples	This study did not use field-collected samples.
Ethics oversight	The Committee of the Animal Experimentation Center at the Sapporo Medical University School of Medicine approved all animal protocols.

Note that full information on the approval of the study protocol must also be provided in the manuscript.

Human research participants

Policy information about studies involving human research participants

Population characteristics	Six wound tissue samples from five diabetic patients (age: 68.7±9.0 years old; HbA1c(NGSP): 7.02±2.15%) and six wound tissue samples from six non-diabetic patients (age: 58.0±21.7 years old) were used in the study. The tissues used in the study were debrided and disposed during surgery for treatment, and no additional excisions were made for the study.
Recruitment	Samples were recruited after agreement was obtained through informed consent.
Ethics oversight	The Ethical Review Board at the Sapporo Medical University in Japan approved the study.

Note that full information on the approval of the study protocol must also be provided in the manuscript.
